# Effects of Composite Supplement Containing Astaxanthin and Sesamin on Cognitive Functions in People with Mild Cognitive Impairment: A Randomized, Double-Blind, Placebo-Controlled Trial

**DOI:** 10.3233/JAD-170969

**Published:** 2018-03-27

**Authors:** Naoki Ito, Hitomi Saito, Shinobu Seki, Fumitaka Ueda, Takashi Asada

**Affiliations:** a Pharmaceutical and Healthcare Research Laboratories, Research and Development Management Headquarters, FUJIFILM Corporation, Ashigarakami-gun, Kanagawa, Japan; b Memory Clinic Ochanomizu, Bunkyo-ku, Tokyo, Japan

**Keywords:** Astaxanthin, CNSVS, cognitive functions, mild cognitive impairment, sesame extract, sesamin

## Abstract

**Background:**

Dementia and its first or transitional stage, mild cognitive impairment (MCI), is a major concern for the aging Japanese society. Thus, the use of dietary supplements to improve or maintain cognitive function has become a topic of public interest.

**Objective:**

In this study, we evaluated the effects of a composite supplement containing food-derived antioxidants, specifically astaxanthin and sesamin (AS), on cognitive function in people with MCI.

**Method:**

Twenty-one healthy participants with MCI were recruited in our double-blind placebo-controlled pilot study. They were assigned to either an AS group, who received ingestible capsules containing AS, or a placebo group, who received identical placebo capsules. To assess cognitive functions, we performed the Japanese version of the Central Nervous System Vital Signs (CNSVS) test and the Alzheimer’s Disease Assessment Scale-Cog test at baseline, after 6 weeks, and after 12 weeks of dietary supplementation.

**Results:**

The CNSVS test revealed significant improvements in psychomotor speed and processing speed in the AS group compared with the placebo group, suggesting that the daily supplementation of AS improved cognitive functions related to the ability to comprehend, and perform complex tasks quickly and accurately.

**Conclusion:**

Our results provide support for the use of AS as a dietary supplementation for improving cognitive functions.

## INTRODUCTION

According to the World Health Organization, approximately 47 million people worldwide suffer from dementia, with 9.9 million new cases every year [1]. Alzheimer’s disease (AD) is the most common cause of dementia. AD is a neurodegenerative disease with many characteristic and pathological features, though the exact causes and mechanisms underlying AD progression are still controversial. Cognitive impairments that are insufficiently severe to warrant a diagnosis of dementia are referred to as mild cognitive impairment (MCI). MCI is described as the first stage or the transitional stage of dementia, because people with MCI develop AD at a rate of 10–15% every year [2]. Numerous new treatments are being investigated, and these are in various stages of clinical trials. However, to date, no effective cures for AD or MCI have been established.

Oxidative stress is thought to be involved in the pathogenesis and progression of age-related cognitive impairments [3]. Because anti-oxidative capacity decreases with age [4], the prevention and treatment of cognitive impairments by food-derived antioxidants are highlighted [5]. Astaxanthin is a red carotenoid found in salmon, shrimp, crab, and microalgae [6, 7]. Astaxanthin exerts a strong anti-oxidative effect by scavenging free radicals such as singlet oxygen [8]. Ingested astaxanthin is absorbed in the small intestine, and reaches the plasma and erythrocytes [9], epidermis and dermis [10], and brain by crossing the blood-brain barrier [11]. The strong anti-oxidative activity of astaxanthin has inspired researchers to evaluate the beneficial effects of astaxanthin as a supplement for wide variety of human health factors including skin conditions [12], metabolism [13, 14], exercise performance [15], and sleep efficiency (in combination with zinc) [16]. The beneficial effects of astaxanthin for brain function have been investigated in both mice and humans [17–19]. Astaxanthin has been found to show neuroprotective effects. Specifically, it reduces oxidative stress in the brain, and is thought to alleviate oxidative stress-related brain dysfunctions. Sesame is a major component in Asian cuisine [20]. Similar to astaxanthin, treatment with sesamin, which is a major lignan found in sesame extract, also produced neuroprotective effects, and has been found to alleviate the cerebral ischemia-induced damage [21, 22].

The beneficial effects of astaxanthin for people with dementia or MCI are still controversial though few studies have evaluated the effects of dietary supplementation with astaxanthin on cognitive function in humans. The effects of astaxanthin on the improvement of cognitive function have been previously evaluated by other group [17]. Although differences between supplementation with astaxanthin and that with placebo were not observed, statistically significant improvement of cognitive function from baseline by supplementation with astaxanthin was observed, which encouraged us to evaluate the combined supplementation with some ingredient which would enhance the effect of astaxanthin. Furthermore, to our knowledge, the effects of sesamin on cognitive function have not been investigated in humans. Several lines of evidence have shown that the absorption and tissue distribution of astaxanthin is different from that of sesamin, suggesting that the beneficial effects of the two compounds are not equivalent [23–25]. Astaxanthin was absorbed with peak plasma concentration at 5 hours [26], while sesamin and its metabolite was absorbed with relatively quick peak at one hour [24]. We hypothesized that the quickly absorbed sesamin reduced the systemic oxidative stress, which could protect astaxanthin from the immediate degradation and eventually brought out much stronger anti-oxidative capacity. In this study, we evaluated the combined effects of dietary supplementation with astaxanthin and sesamin (AS) on a wide range of cognitive functions in people with MCI. We used the Japanese version of the Central Nervous System Vital Signs (CNSVS, also known as ‘Cognitrax’ in Japan) computerized battery test [27] and the Alzheimer’s Disease Assessment Scale-Cog (ADAS-Cog) as the primary outcomes [28]. As secondary outcomes, we examined blood elements related to oxidative stress and brain-derived neurotrophic factor (BDNF) levels, and also conducted a safety evaluation.

## MATERIALS AND METHODS

### Study design, randomization, and blinding

This study was a randomized, double-blind, placebo-controlled, parallel-group comparison trial conducted to evaluate the effects of dietary supplementation with AS on cognitive function in MCI participants. We allocated an equal number of participants to the placebo and active groups. The study was approved by the Ethical Committee of Nihonbashi Cardiology Clinic (Approved Number: NJI-016-07-01). This study was registered in the UMIN Clinical Trials Registry (ID: UMIN000023391). The study protocol adhered to the Declaration of Helsinki and the Ethical Guidelines for Medical and Health Research Involving Human Subjects. Participants, practitioners, and clinicians were blinded. Clinicians performed the intervention and outcome measurements. Practitioners performed the analysis. As long as we examined, the minimum number of participants required to evaluate the intervention effects using the CNSVS test was 10 [29]. According to our previous independent trials conducted on 10 healthy people [30], we set the required sample size as 10. We set the CNSVS and ADAS-cog tests as the primary outcomes. We also set the safety evaluation and the blood test for malondialdehyde (MDA), oxidized low-density lipoprotein (ox-LDL), serum total antioxidant status (STAS), paraoxonase 1 (PON-1), and BDNF as the secondary outcomes. Because eligible participants were serially incorporated into the study, participants were assigned to the AS or placebo group using an alternating quasi-randomization allocation method. Allocation was concealed until all participants finished the tests. The one who was different from the controller of this study enrolled and assigned the participants according to sex, age, and score on the Hierarchic Dementia Scale-Revised (HDS-R). The participants were enrolled by the responsible doctor.

### Participants

This study included participants aged from 50 to 79 years old in the Tokyo metropolitan area who attended Memory Clinic Ochanomizu, and were diagnosed with MCI. Participants who had 24 to 27 points of Mini-Mental State Examination Japanese Version scores were diagnosed with amnestic MCI [31]. Participants were confirmed to have no arteriosclerotic dementia with magnetic resonance imaging or computed tomography scans performed within the previous 6 months. Each participant provided written informed consent after receiving a detailed explanation regarding the objectives and procedure of the study. This study consisted of a 12-week supplement ingestion period from August to December 2016. Participants with the following criteria were excluded from the study: 1) individuals who were taking medicine for cognitive impairment, 2) individuals who were taking supplements for improvement of cognitive function, 3) individuals who had been diagnosed with dementia according to the HDS-R, 4) individuals who had been diagnosed with depression according to the Japanese version of the Geriatric Depression Scale Short Form (GDS-S-J), 5) individuals who had impaired color vision, 6) individuals who had unequal function in the right and left hands due to injury or surgery, 7) individuals who were included or intended to participate in other trials which performed the oral administration of supplement or medicine, or percutaneous treatment with cosmetic or medicine, or who had participated in these kind of trials within one month, 8) individuals who had been classified as ineligible for this study by responsible doctor.

### Supplement formulation

One AS capsule contained 3 mg of astaxanthin, 5 mg of sesamin, and other components including filling agents such as safflower oil, and dispersants. The placebo group received a placebo capsule that contained filling agents such as safflower oil, starch and water, dispersants, artificial colorants, but did not contain AS. Two capsules were administered every day for 12 weeks. AS capsules and placebo capsules were not distinguishable by their shape, taste, or color. The AS capsules contained natural astaxanthin derived from *Haematococcus pluvialis* (ASTOTS, FUJIFILM), which was processed using the dispersant technology that has been found to improve the absorbability of astaxanthin in humans [26]. We used sesame extract derived from *Sesamum indicum*. The dose of astaxanthin was determined by the previously performed study by other group, which evaluated the effect of 6 mg or 12 mg of astaxanthin on cognitive function in humans [17]. We decided the dose of sesamin by referring the previous study from another group, which evaluated the effect of 10 mg of sesamin on fatigue in humans [32].

### Cognitive tests

The CNSVS is a computerized test that evaluates multiple cognitive functions. CNSVS scores have been standardized according to the results from large populations of individuals aged 7–90 years [27]. The test can detect subtle cognitive changes that occur during aging or MCI [27, 33]. It evaluates 11 neuropsychological domains (composite memory, verbal memory, visual memory, processing speed, psychomotor speed, executive function, reaction time, complex attention, simple attention, cognitive flexibility, and motor speed) using 7 measures: verbal and visual memory tests, the finger tapping test, the symbol digit coding (SDC) test, the Stroop test, the shifting attention test, and the continuous performance test. In the finger tapping test, participants pressed the space key of a keyboard with their right or left index finger as many times as they could within 10 seconds. This test is known as a sensitive neuropsychological test that is used to identify brain impairments [27]. In the SDC test, the participants were given 120 seconds to type numbers that corresponded to a series of symbols shown on a screen. The participants first completed a training session in which they learned the link between the numbers and symbols by viewing a table showing the 8 symbols above each of the 8 numbers. This table was in plain sight throughout the SDC test. In the trial, they typed numbers in empty boxes on the screen that corresponded to the symbols. This test is thought to be highly sensitive to cerebral dysfunction [27]. The details of the other tests are described in elsewhere [27]. The CNSVS test was performed at baseline and after 6 and 12 weeks of dietary supplementation.

The Japanese version of the ADAS-cog is one of the most popular tests of cognitive function [34]. The test is composed of 11 tasks that measure impairments in memory, language, praxis, attention, and other cognitive functions related to symptoms of AD. The ADAS-cog test was performed at baseline and after 6 and 12 weeks of supplementation.

### Blood sampling and safety evaluation

Serum and plasma were obtained from the participants at baseline, after 6 weeks, and after 12 weeks of dietary supplementation. MDA, ox-LDL, STAS, PON-1, and BDNF were analyzed by NIKKEN SEIL Co, Ltd for each time point. As a safety evaluation, we performed a general biochemical examination of blood and hematologic characteristics.

### Statistical analysis

All results were presented as the mean±standard deviation (SD). The intra-group change from the baseline was evaluated using Dunnett’s test. Differences between the placebo and AS groups were assessed by unpaired *t*-test. No additional analyses were performed. Probabilities less than 5% (^*^p < 0.05) were considered to be statistically significant. Statistical analyses were carried out using IBM SPSS statics (version 24).

## RESULTS

### Participants

**Fig.1:**
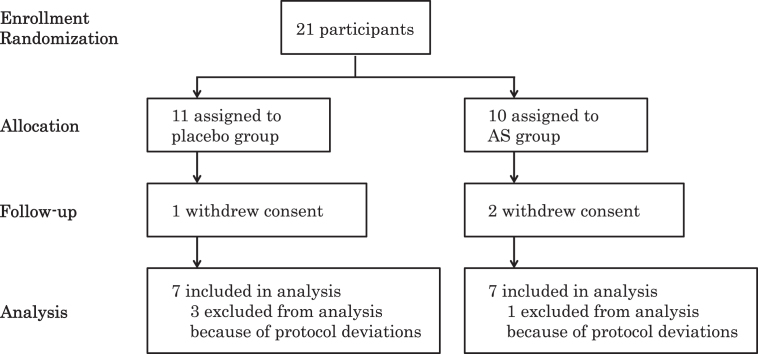
Flow diagram of participants. AS, astaxanthin and sesamin.

Initially, we recruited 21 participants (aged 57–78, 14 males and 7 females) in the Tokyo metropolitan area who had been diagnosed with MCI. The included participants were assigned to the AS group (*n* = 10) or placebo group (*n* = 11). One participant in the placebo group and two participants in the AS group discontinued the study due to withdrawal of consent for personal reasons. Three participants in the placebo group and one participant in the AS group were excluded from the analysis due to deviations from the protocol (Fig. 1). Finally, we analyzed data from 14 participants (aged 57–78, 9 males and 5 females). We performed per-protocol set analysis. There were no statistical differences between the initial and final groups of participants in terms of baseline data regarding age, BMI, GDS-S-J, and HDS-R. The participants were recruited from August to September 2016. This study consisted of 12-week administration period from August to December 2016. The placebo group and AS group were matched according to age, gender, and BMI (Table 1). We observed no differences in baseline GDS-S-J and HDS-R scores between the placebo and AS groups. The average ingestion rates were not significantly different: 99.7±1.5% and 97.8±2.2% in the placebo and AS group, respectively. All subjects had an ingestion rate greater than 95%.

**Table 1:** Baseline characteristics of participants who completed 12 weeks test

	Placebo (*n* = 7)	AS (*n* = 7)	*p* value
Age (mean±SD)	68.4±7.3	70.4±7.0	0.61
Male, *n* (%)	4 (57.1)	5 (71.4)
BMI (mean±SD)	22.1±2.7	21.6±3.4	0.76
GDS-S-J (mean±SD)	5.43±3.7	3.0±2.6	0.18
HDS-R (mean±SD)	28.4±1.5	29.3±1.1	0.25

### Cognitive tests

**Fig.2:**
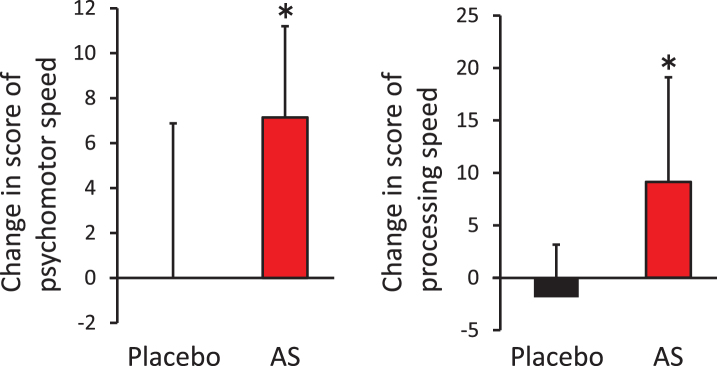
Improvement of psychomotor speed and processing speed following dietary supplementation with astaxanthin and sesamin. Change in psychomotor speed (left) and processing speed (right) from baseline. ^*^p < 0.05 by unpaired *t*-test. Error bars indicate SD. AS, astaxanthin and sesamin.

The aim of this study was to evaluate the effects of dietary supplementation with AS on cognitive functions. We performed the CNSVS and ADAS-cog tests at baseline, after 6 weeks, and after 12 weeks of supplementation. For the CNSVS test, we observed no statistically significant differences in the raw values between the AS and placebo groups (Table 2). However, the individuals in the AS group showed a significantly greater amount of change between the baseline and 12 weeks after supplementation in terms of psychomotor speed and processing speed, compared with individuals in the placebo group (Fig. 2). Furthermore, in the AS group, we observed a statistically significant increase in processing speed between the baseline and 12 weeks post-supplementation (*p* = 0.018), although we observed no statistical difference in psychomotor speed (*p* = 0.133). For the ADAS-cog test, we observed no differences between the placebo and AS groups at any time points.

**Table 2:** Score of cognitive tests

CNS Vital Signs domain scores	Week		
		0	6	12
	Group	mean±SD	mean±SD	mean±SD
Composite Memory	Placebo	94.0±16.7	100.0±13.2	93.7±16.6
	AS	98.1±17.9	94.6±16.2	89.0±12.9
Verbal Memory	Placebo	93.7±17.2	98.0±17.7	92.7±16.5
	AS	92.3±14.1	88.4±14.9	90.0±17.0
Visual Memory	Placebo	96.0±17.4	103.1±9.0	97.4±13.7
	AS	105.0±17.3	103.4±15.2	91.9±7.5
Psychomotor Speed	Placebo	104.0±14.1	102.3±15.6	104.0±9.3
	AS	102.0±15.0	102.7±17.4	109.1±15.1
Reaction Time	Placebo	87.0±17.8	93.7±16.1	91.0±11.1
	AS	83.9±13.6	83.6±15.2	86.7±13.3
Complex Attention	Placebo	99.3±10.8	97.1±12.9	109.1±11.5
	AS	102.9±10.4	104.0±6.6	106.4±10.7
Cognitive Flexibility	Placebo	91.7±12.4	92.7±10.2	102.7±11.9
	AS	98.3±8.3	98.0±7.4	101.9±9.1
Processing Speed	Placebo	111.9±10.3	111.0±14.0	110.0±8.4
	AS	105.7±14.6	112.4±13.9	114.9±16.1
Executive Function	Placebo	91.0±13.0	94.6±11.7	102.3±12.1
	AS	98.4±7.9	97.9±7.7	102.6±8.7
Simple Attention	Placebo	102.9±8.0	96.9±18.3	104.3±7.2
	AS	101.7±14.7	103.0±14.1	98.9±15.6
Motor Speed	Placebo	96.6±14.6	95.1±13.4	98.0±11.3
	AS	98.7±11.8	95.9±15.0	102.1±10.4
ADAS-cog	Placebo	3.00±1.75	3.29±1.86	2.27±1.22
	AS	5.61±3.26	4.27±2.76	3.89±3.26

### Blood tests

Extensive epidemiological research has demonstrated an association between the risk of cognitive decline and systemic oxidative stress [35]. To address this, we analyzed blood levels of MDA, ox-LDL, STAS, and PON-1 at baseline, after 6 weeks, and after 12 weeks of supplementation. Higher serum BDNF levels were suggested to protect against future occurrence of dementia and AD [36]. Because administration of astaxanthin is known to alleviate oxidative stress-induced decreases in BDNF levels, we also analyzed blood levels of BDNF at baseline, after 6 weeks, and after 12 weeks of dietary supplementation [37]. We excluded the MDA results from the consideration because the statistical trend between the placebo and AS groups at baseline was observed (0.87±0.29 in placebo group versus 0.63±0.10 in AS group, *p* = 0.054). We did not observe statistically significant differences between the placebo and AS groups for ox-LDL, STAS, PON-1, or BDNF for any time points (data not shown).

### Clinical safety

We observed several adverse events in both the placebo and AS groups (Table 3). There were no differences in the frequency of adverse events between the groups. We did not observe any adverse events related to the ingestion of AS. We found some statistically significant changes in the scores from the general biochemical blood examination and hematologic tests. However, all of these changes were slight and well within normal values. The responsible doctor confirmed that they did not represent a safety concern.

**Table 3:** List of adverse events

	Placebo	AS
Acute low back pain	1	0
Cold	0	3
Cystitis	1	0
Diarrhea	0	1
Dizziness	0	1
Feeling of smothering	2	0
Lassitude	2	0
Lip redness	0	1
Low back pain	1	0
Malaise	1	0
Occult blood in urine	0	1
Protein in urine	0	1
Slight cold	0	1
Sore throat	0	1
Stomatitis	0	1
Tonsillitis	1	0

## DISCUSSION

We found that dietary supplementation with AS for 12 weeks improved psychomotor speed and processing speed, as measured by the CNSVS test, which was related to the ability to comprehend, and perform complex tasks quickly and accurately [38, 39]. Psychomotor speed is calculated as the sum of the finger tapping test and total correct responses in the SDC test. This reflects “How well a subject perceives, attends, responds to complex visual-perceptual information and performs simple fine motor coordination”, and is related to “Ability preform simple motor skills and dexterity through cognitive functions, i.e., use of precision instruments or tools, performing mental and physical coordination, i.e., driving a car, playing a musical instrument” [27, 38]. Processing speed is calculated by subtracting the number of errors from the correct responses in the SDC test. Processing speed reflects “How well a subject recognizes and processes information i.e., perceiving, attending/responding to incoming information, motor speed, fine motor coordination, and visual-perceptual ability”, and is related to “Ability to recognize and respond/react, i.e., fitness-to-drive, occupation issues, possible danger/risk signs or issues with accuracy and detail” [27, 38]. The majority of clinical trials evaluating the effects of supplements on cognitive function have focused on memory improvement. Thus, the lack of improved verbal memory or visual memory observed in the AS group may indicate a unique effect of AS dietary supplementation on cognitive function, distinguishing it from other supplements such as *Ginkgo Biloba*. Although astaxanthin can cross the blood-brain barrier [11], its detailed distribution in the brain is unknown. Because carotenoids show inclined distribution in some tissue [41], analysis of the detailed distribution of astaxanthin and its bioactivity in the brain merits future investigation to explain the unique role of AS on cognitive function, because both psychomotor speed and processing speed are related to frontal lobe function. Recently, the presence of brain amyloids in earlier stages of MCI was associated with increased safety risks of driving [42]. Because psychomotor speed is related to mental and physical coordination, which are required for using some instrument or driving a car [39, 43], AS dietary supplementation might be useful for reducing the risk of car accidents in the aging Japanese population. In addition, AS supplementation will improve the ability of elderly people to quickly and accurately judge complex information, which is related to processing speed.

To our knowledge, this study is the first to examine the effects of combined supplementation of AS on cognitive functions. The effects of dietary supplementation with astaxanthin only on the improvement of cognitive functions has been previously performed using Coghealth, although statistically significant differences between the placebo and astaxanthin groups were not observed [17]. As described in the Materials and Methods section, we used the dispersant technology to enhance the absorbability of astaxanthin in humans [26]. This technology appears to have reinforced the effects of astaxanthin on cognitive functions. Indeed, astaxanthin has a neuroprotective effects in that it reduces oxidative stress, and has been found to alleviate oxidative stress-related brain dysfunctions, improve learning or memory, prevent neurodegeneration, cellular toxicity, or apoptosis of neurons, and promote cellular survival [17–19, 44–46]. In addition to neuroprotective effects, administration of astaxanthin enhanced adult hippocampal neurogenesis and spatial memory in mice [47]. Administration of astaxanthin also prevented depression by inhibiting hippocampal inflammation in diabetic mice [48]. Furthermore, the activities of endogenous anti-oxidative enzymes were upregulated by treatment with astaxanthin, suggesting that astaxanthin acted as antioxidant both by anti-oxidative capacity itself and by activating endogenous anti-oxidative capacity [49]. In addition to astaxanthin, administration of sesamin has been found to have the neuroprotective effects in cerebral ischemia or traumatic brain injury [21, 22, 50]. Sesamin also prevented the inflammation in co-culture of microglia and neuron [51], or protect PC12 cells from high glucose-induced oxidation and apoptosis [52]. Sesamin and its metabolites are also known to promote neuronal differentiation [53]. These pleiotropic effects of AS might be involved in the improvement of cognitive function. In addition to the independent effects of astaxanthin or sesamin, we hypothesized that the cognitive functions were improved by the synergistic effects of AS, as described in Introduction section. The between-group differences observed in this study reinforced our hypothesis. However, the effects of sesamin on cognitive function have not been investigated in humans. Thus, we could not conclude that the improvement of cognitive functions observed in this study was derived from astaxanthin with enhanced absorbability, sesamin, or synergistic effects of AS.

A previous extensive epidemiological study showed that high levels of thiobarbituric acid-reactive substances that reflected the level of lipid peroxidation were correlated with the risk of cognitive decline [35]. The level of lipid peroxidation in human erythrocytes has been found to be reduced by administration of astaxanthin [9]. Furthermore, astaxanthin is known to cross the blood-brain barrier, and exert a neuroprotective effect by reducing oxidative stress or increasing the BDNF level [11, 37, 54]. We therefore expected administration of AS to decrease systemic levels of oxidative stress, and to increase levels of BDNF. Although we did not observe systemic changes in STAS, PON-1, or BDNF levels, reduced oxidative stress or increased levels of BDNF in microenvironments in the brain might have been involved in the observed improvement of cognitive functions.

Our study has several limitations. Even though our sample size was limited and we performed per-protocol set analysis which rated this trial as pilot study, we observed AS-induced improvements in psychomotor speed and processing speed, suggesting that AS have strong effects on cognitive functions. Astaxanthin is found in foods such as shrimp, salmon, and crab. These types of seafood, along with sesame, are important elements of Japanese cuisine. In this study, we did not estimate participant dietary intake of astaxanthin and sesame, leading to a possible over- or under-estimation of the effects of AS. We hypothesized that combined supplementation of AS exerted much stronger anti-oxidative activity than single supplementation. In fact, we observed the improvement of cognitive functions. However, administration of AS led to no observable changes in systemic levels of oxidative stress or BDNF. Thus, precise mechanisms how AS improved cognitive functions were not elucidated. Further analysis is required to examine how AS influence cognitive functions.
